# Advances in Acute Myeloid Leukemia: Recently Approved Therapies and Drugs in Development

**DOI:** 10.3390/cancers12113225

**Published:** 2020-11-01

**Authors:** Michele Stanchina, Deborah Soong, Binbin Zheng-Lin, Justin M. Watts, Justin Taylor

**Affiliations:** 1 Department of Medicine, Sylvester Comprehensive Cancer Center, University of Miami Miller School of Medicine, Miami, FL 33136, USA; michele.stanchina@jhsmiami.org (M.S.); deborah.soong@jhsmiami.org (D.S.); 2 Department of Pediatrics, University of Miami Miller School of Medicine, Miami, FL 33136, USA; 3 Department of Medicine, Icahn School of Medicine Mount Sinai West-Morningside, New York, NY 10025, USA; Binbin.Zheng@mountsinai.org; 4 Division of Hematology, Department of Medicine, Sylvester Comprehensive Cancer Center, University of Miami Miller School of Medicine, Miami, FL 33136, USA; jxw401@miami.edu

**Keywords:** novel therapeutics AML, FLT3, IDH1/2, CD33, BCL2, CD47, CD123, hedgehog, anti-body drug conjugate, immunotherapy

## Abstract

**Simple Summary:**

Acute myeloid leukemia (AML) is one of the most common types of leukemia in adults, with an average first diagnosis at age 68, and has historically carried poor prognosis due to various genetic alterations and abnormalities that complicate approaches to treatment. Recently, numerous advancements have been made within the realm of AML therapy, including genetically targeted therapies against FLT3, IDH1/2 and tumor protein p53 (TP53)*,* antibody-drug conjugates, and immunotherapies. Alongside these developments in targeted therapies, we have acquired a better understanding of mechanisms of resistance against conventional therapies that have been in use for decades. The goal of our review is to serve as a guide in the latest targeted and immunotherapeutic drugs available, as well as those currently in the pipeline. We review their specific mechanisms, their characteristic properties, indications for use, outcomes in efficacy in clinical trials prior to approval by the FDA, and their usage in combination with other available therapies.

**Abstract:**

Acute myeloid leukemia (AML) is a genetically heterogeneous malignancy comprised of various cytogenetic and molecular abnormalities that has notoriously been difficult to treat with an overall poor prognosis. For decades, treatment options were limited to either intensive chemotherapy with anthracycline and cytarabine-based regimens (7 + 3) or lower intensity regimens including hypomethylating agents or low dose cytarabine, followed by either allogeneic stem cell transplant or consolidation chemotherapy. Fortunately, with the influx of rapidly evolving molecular technologies and new genetic understanding, the treatment landscape for AML has dramatically changed. Advances in the formulation and delivery of 7 + 3 with liposomal cytarabine and daunorubicin (Vyxeos) have improved overall survival in secondary AML. Increased understanding of the genetic underpinnings of AML has led to targeting actionable mutations such as *FLT3*, *IDH1/2* and *TP53*, and BCL2 or hedgehog pathways in more frail populations. Antibody drug conjugates have resurfaced in the AML landscape and there have been numerous advances utilizing immunotherapies including immune checkpoint inhibitors, antibody-drug conjugates, bispecific T cell engager antibodies, chimeric antigen receptor (CAR)-T therapy and the development of AML vaccines. While there are dozens of ongoing studies and new drugs in the pipeline, this paper serves as a review of the advances achieved in the treatment of AML in the last several years and the most promising future avenues of advancement

## 1. Introduction

Acute myeloid leukemia (AML) is a complex disease characterized by clonal expansion of undifferentiated myeloid precursors in the bone marrow and resultant failed hematopoiesis. Recent advances in sequencing methodologies and functional experimentation have allowed us to understand the role of genomic aberrations in AML pathogenesis. Subsequently, AML has emerged as a dynamic disorder in which multiple sub-clones coexist and compete with each other, not only during the natural course of disease but also under selective pressure created by antineoplastic agents [1,2]. Moreover, after decades of relative stagnation in therapeutic progress, the treatment of AML is changing at an unprecedented pace due to rapid advances in genetics, understanding of molecular pathogenesis and development of novel therapeutics. For those in clinical practice it is imperative to stay abreast of all the new treatments available and those in the pipeline. This review covers the actionable genetic mutations in AML and the corresponding key therapeutic trials targeting these alterations, as well as novel therapies that are not specific for individual mutations. Finally, we overview the drug development landscape focusing on some of the most interesting and promising treatments in clinical trials.

## 2. Recently Approved Agents in AML

Traditional chemotherapy treatments consist of either a high dose regimen containing cytarabine for 7 days and an anthracycline given for 3 days (7 + 3), or lower-intensity treatments with a hypomethylating agent (HMA) such as azacitidine (AZA) or decitabine, or low dose cytarabine (LDAC) [3]. Mitoxantrone can also be used interchangeably with anthracyclines [4]. While standard 7 + 3 induces remission in up to 60% of individuals, intensive chemotherapy is often not a viable option for certain patients based on advanced age, poor baseline functional status, significant co-morbidities or organ dysfunction [5]. While age itself is not a specific reason to defer intensive chemotherapy, older patients in general have more high-risk features, such as AML with myelodysplasia-related changes (AML-MRC) therapy-related AML (t-AML), complex karyotype or *TP53* mutation [6], and are less likely to respond to chemotherapy. While new therapies were needed to improve on the standard intensive chemotherapy regimens, more effective lower intensity regimens were sorely needed for older patients. Fortunately, advances in molecular and genetic understanding and testing, along with new drug approvals have significantly changed the treatment landscape of AML. Below, we review the new approvals in AML based on genetic targets of therapy, followed by non-genetic targets and immunotherapy. These approvals and their indications are listed in Table 1.

## 3. Genetically Targeted Therapies

### 3.1. FLT3

Approximately half of patients with de-novo AML have a normal karyotype (NK-AML) on conventional cytogenetics [7] and would be considered intermediate-risk. Genomic findings permit further stratification of NK-AML into high- or low-risk groups. Compared to other adult malignancies, AML has one of the lowest mutational burdens with an average number of 13 coding mutations per patients, with five of them being recurrent mutations [8]. FMS-related tyrosine kinase 3 gene (*FLT3*) is one of the most highly recurrently mutated genes in AML and one of the earliest discovered [9,10]. This gene encodes a transmembrane tyrosine kinase receptor that is expressed in early hematopoietic stem cells (HSC) and progenitor cells and stimulates cell proliferation upon activation. Mutations in *FLT3* are present in around 30% of newly diagnosed cases of AML, nonetheless, *FLT3* aberrations are neither necessary nor sufficient for AML development [11,12]. *FLT3* mutations are considered to be secondary, or driver mutations, as both subtypes of *FLT3* mutation can generate proteins that will allow them to spontaneously dimerize and thus lead to activation of multiple downstream pathways promoting rapid proliferation [13]. The majority of *FLT3* mutations are internal tandem duplications (ITD) which result in a receptor that activates in a ligand-independent manner [14], and predicts poor prognosis due to a high relapse rate, lower overall survival (OS) and more aggressive disease [15,16]. The less common mutations in the tyrosine kinase domain (TKD) hold uncertain prognosis [17,18].

Midostaurin, is an oral multi-targeted kinase inhibitor with activity against activated FLT3 that has altered the landscape for treating de-novo AML. The Cancer and Leukemia Group B (CALGB) 10,603 RATIFY trial was a multi-institutional, multinational, randomized, double-blind placebo-controlled trial that examined the effects of adding midostaurin to standard chemotherapy in adults aged 18–59 years old with newly diagnosed AML and an *FLT3* mutation. Patients received standard 7 + 3 induction chemotherapy and then received either midostaurin or placebo on days 8–21. Based on a day 21 bone marrow biopsy, if patients achieved complete remission (CR), they underwent consolidation chemotherapy with midostaurin, administered on days 8–21 for four cycles, and then entered a maintenance phase with midostaurin or placebo. The rate of CR was 58.9% in the midostaurin group and 53.5% in placebo. Median OS was 74.7 months in the midostaurin group and 25.6 months in the placebo, with a hazard ratio (HR) of 0.78 (95% CI, 0.63 to 0.96; *p* = 0.009). OS was consistent in *FLT3* groups—ITD (high and low ratio of mutant allele) and TKD [17]. Median event free survival (EFS) was longer in the midostaurin group (8.2 months) vs. placebo (3 months), and patients in the midostaurin group had a 21.6% lower likelihood of having an event, including death from any cause or failure to achieve CR. Median disease-free survival (DFS) was also increased with midostaurin to 26.7 months up from 15.5 months with placebo [17]. Overall, the results of the CALGB-RATIFY trial were practice changing, showing that younger adults with newly diagnosed AML and an *FLT3* mutation had a 22% lower risk of death with the addition of midostaurin to standard chemotherapy. Midostaurin was FDA-approved for use in combination with chemotherapy in April 2017 and is now considered the standard of care for patients with de novo *FLT3*-mutant AML.

In the CALGB-RATIFY trial (NCT00651261), hematopoietic stem cell transplantation (HSCT) was not part of the protocol but was allowed at the discretion of the investigator. HSCT was performed during first remission in more patients receiving midostaurin than placebo, thus potentially biasing the results in favor of midostaurin [17]. The assumption surrounding this data has been that patients on the midostaurin arm likely had lower levels of measurable residual disease (MRD) and in general, patients with morphologic remission with no detectable MRD prior to transplant have better outcomes than patients with detectable MRD or morphologic persistent disease [19]. Therefore, midostaurin may have been more effective in inducing MRD negativity, but that has not yet been studied prospectively. Assays to detect MRD have been difficult to develop due to the heterogeneity of the disease. Levis et al. developed a highly sensitive assay combining PCR with next generation sequencing (NGS) to detect MRD. They validated the assay using a subset of 17 patients with *FLT3*-ITD, nucleophosmin-1 mutations (*NPM1*) mutations and intermediate risk karyotypes. All patients received chemotherapy, and eight received FLT3-inhibitors. All achieved remission by flow cytometry and PCR and then underwent HSCT. The average level of ITD mutations was significantly lower in the group that had received an FLT3-inhibitor, suggesting that the use of an FLT3-inhibitor in addition to chemotherapy may result in a deeper remission and better prognosis post-transplant [19]. Using the *FLT3* mutation to monitor MRD is highly specific, however, the downside is that *FLT3* can be lost at relapse, or only present at relapse. This novel combined PCR-NGS assay is promising, but currently has only been validated in *FLT3* with *NPM1* mutations and may hold different value in patients with more adverse genotypes. This assay is currently being studied with chemotherapy plus quizartinib, as well as gilteritinib in post-transplant maintenance therapy [19].

Numerous second-generation FLT3 tyrosine kinase inhibitors (TKIs) have now been developed designed to more specifically target FLT3. Type I FLT3 TKIs including sunitinib, midostaurin, crenolanib, and gilteritinib, bind both active and inactive receptors in the ATP binding pocket and can target both ITD and TKD mutations, while type II TKIs such as sorafenib, ponatinib and quizartinib only bind inactive FLT3 and do not target TKD mutations [13]. Gilteritinib, is the only second-generation FLT3 TKI to gain FDA-approval and was approved in November 2018 as a single agent for use in relapsed or refractory (R/R) AML. In a phase I/II trial, gilteritinib monotherapy resulted in sustained *FLT3* inhibition, with 41% of R/R patients achieving CR or CR with incomplete hematologic recovery (CRi). The phase III, multi-centered ADMIRAL trial comparing gilteritinib against salvage chemotherapy in patients with R/R AML and *FLT3* mutations, revealed significantly longer OS (9.3 months vs. 5.6 months), with longer EFS and fewer grade three adverse events (AEs). Patients randomized to chemotherapy received MEC, FLAG-IDA, or low dose HMA. While more people in the gilteritinib-treated group received HSCT than in the chemotherapy group (25.5% vs. 15.3%), the OS benefit was evident even prior to HSCT. More patients achieved CR/CRi in the gilteritinib-treated group than with chemotherapy alone, even when including remission post-transplant. Additionally, patients with *FLT3*-ITD and *FLT3*-TKD mutations responded similarly to gilteritinib, and longer survival was observed for *NPM1* and *DNMT3A* co-mutations. Importantly, baseline levels of AXL did not affect survival rates, implicating that gilteritinib may be an important TKI to use with AXL mutations. Additionally, while *FLT3*-TKD mutations are uncommon at initial presentation, they can emerge after FLT3 TKI therapy and cause secondary resistance, which also supports the use of gilteritinib in R/R FLT3 AML as it targets both ITD and TKD. At the publication of the study, 38 participants were still receiving gilteritinib, with the most common serious AEs being febrile neutropenia, anemia, thrombocytopenia and elevated liver enzymes. Overall, the HR for death was 0.64 which is promising, however, long term survival in both groups still remained poor [12]. Future studies are underway examining the potential role of gilteritinib during induction, consolidation therapy, post-consolidation and post-transplant maintenance therapy.

Crenolanib, a highly potent and specific second generation FLT3 TKI designed to target both ITD and TKD mutations, has also been studied in de novo *FLT3* AML and is effective against D835 mutations, which confer resistance to sorafenib and quizartinib [20]. A small phase II trial in de novo *FLT3* AML in patients 60 or younger examined 7 + 3 induction chemotherapy plus crenolanib, and consolidation chemotherapy followed by either HSCT or maintenance crenolanib therapy. CR was achieved in 24/29 (83%) patients, and after 14 months, only two patients out of the 24 had experienced relapses [21]. These data suggest that adding crenolanib to standard induction chemotherapy in younger patients with *FLT3*-mutated AML may be associated with a high rate of CR and low relapse rate. A phase III trial of 7 + 3 and crenolanib vs. midostaurin is currently in progress.

As previously detailed, second generation FLT3 TKIs, in combination with 7 + 3 chemotherapy, are yielding higher rates of CR/CRi than midostaurin by up to 20–30% [13], but more studies are needed to see the impact on OS. Trials of 7 + 3 with crenolanib, gilteritinib and quizartinib are currently underway [22]. FLT3-inhibitors in combination with HMA are also being examined in older individuals or individuals who are unfit for intensive chemotherapy with de novo AML. Midostaurin + AZA yielded a 26% response rate, gilteritinib + AZA yielded a 60% response rate, while sorafenib + AZA (46%) or sorafenib + decitabine (83%) yielded even higher overall response rates [13].

Additionally, FLT3-inhibitors are increasingly being studied as maintenance therapy after HSCT to reduce the risk of relapse. The SORMAIN trial was a randomized, double-blind, placebo-controlled study in adults with *FLT3* AML who had undergone HSCT and achieved CR, who were randomly assigned to receive sorafenib as maintenance therapy or placebo. Two-year median relapse-free survival (RFS) defined as hematological relapse or death was 53.3% in the placebo and 85% in sorafenib group [23]. Similarly, Midostaurin showed promising results for maintenance therapy in the RADIUS trial, a randomized, open-label, phase II exploratory trial (NCT01883362) investigating whether the addition of midostaurin after HSCT could reduce the risk of relapse in patients with *FLT3*-ITD+ AML. The RADIUS trial built upon the results of the RATIFY trial which showed promising results when midostaurin was added to induction chemotherapy, and that patients undergoing HSCT had the highest chance of sustained remission, yet the relapse rate remained high. The addition of midostaurin in the RADIUS trial showed a 46% relative reduction in relapse over standard of care [24]. The ADMIRAL and the QuANTUM-R trial supported use of *FLT3*- TKI post HSCT in R/R AML, although these were not randomized. Patients in the ADMIRAL trial that resumed gilteritinib after HSCT had a median OS of 16.2 months vs. 8.4 months without maintenance therapy [13]. Ongoing phase III studies are examining placebo vs. gilteritinib as maintenance therapy after transplant (NCT02997202) [13].

Many new FLT3 TKIs are currently in development. SEL24/MEN1703 is a PIM and FLT3 inhibitor and FF-10101-01 is an irreversible FLT3 inhibitor with activity against multiple FLT3 mutants including ITD, ITD-691L and D835 that are currently in phase I trials. MAX-40279 is an FGFR and FLT3 inhibitor active against ITD and D835, also being investigated [13]. Having a broader range of FLT3 TKIs available will give clinicians greater ability to tailor specific pharmaceutical therapeutics to specific mutation combinations and potential sequencing of therapies to prevent/treat resistance mechanisms that may arise.

### 3.2. IDH1/2

Similar to the utilization of targetable mutations such as *FLT3*, pharmacological advances have been made against recurrent mutations in the isocitrate dehydrogenase 1 and 2 genes (*IDH1*, *IDH2*). *IDH1* mutations and *IDH2* mutations occur in 6–10% and 9–13% of patients with AML, respectively [25]. IDH1 and IDH2 are metabolic enzymes that normally catalyze the conversion of isocitrate to α-ketoglutarate, however, mutations in *IDH1* and *IDH2* encode neo-morphic enzymes that instead reduce α-ketoglutarate to R-2-hydroxyglutarate (2HG), an onco-metabolite that promotes cytokine-independent cell proliferation with blocked differentiation [26,27]. These genetic modifications can also lead to increased epigenetic alterations including histone and DNA methylation which can impair differentiation, as well as other chromatin modifications, altered response to hypoxia, and increased leukemogenic effects by inducing dependence on B-cell lymphoma 2 (BCL-2) [3,28,29]

While mutant *IDH* is associated with poor prognosis in other malignancies, its prognostic value is inconsistently defined in AML, which may be the result of a complex gene–gene interaction [30,31,32]. For instance, *IDH2*-R140 shows strong co-mutation rate with *NPM1*, while *IDH2*-R172 occurs independently of *NPM1* and other class-defining lesions [32]. Notably, this later mutated form is found in only 1% of patients and constitutes an independent subgroup with a unique gene-expression and CpG methylation profile (cytosine followed by guanine residues) as well as prognostic significance [32].

Ivosidenib and enasidenib are oral, mutant-selective small-molecule inhibitors of IDH1 and IDH2, respectively. Of the two, enasidenib was first to be approved. In pre-clinical studies, enasidenib’s inhibition of mutant IDH2 decreased total serum 2-hydroxyglutarate (2-HG) by more than 90%, reducing abnormal histone hypermethylation, and restoring cell differentiation. Further studies from ex-vivo bone marrow of patients with IDH2 mutant AML, showed that enasidenib induced the maturation of blasts to fully functional neutrophils [28]. In fact, there was a dose-dependent survival advantage in xenotransplant models with primary AML treated with enasidenib. In a phase I/II study in R/R AML in patients with *IDH2* mutation (NCT01915498), enasidenib showed promising results.

In the first phase of the trial, dose limiting toxicities along with pharmacokinetic and pharmacodynamic profiles were assessed. This was then followed by a dose escalation phase and an expansion phase which was comprised of four different cohorts—patients >60 years old with R/R disease, any age with R/R AML post HSCT, <60 years with R/R AML without prior HSCT, or >60 years with untreated AML who were ineligible for high dose chemotherapy. Overall 239 patients, 113 in the dose-escalation phase and 124 in the expansion phase were evaluated. Enasidenib exhibited high dose-proportional plasma exposure, rapidly reaching steady state by cycle two day 1. A dose of 100 mg was selected for study expansion for its pronounced reduction in 2-HG plasma levels during phase I of the study. Grade three and four toxicities included hyperbilirubinemia and differentiation syndrome. Hyperbilirubinemia was not associated with transaminitis and was not thought to indicate intrinsic liver toxicity but perhaps an effect similar to Gilbert syndrome where inhibition of the UGT1A1 enzyme leads to decreased bilirubin metabolism. It was also hypothesized that enasidenib could be associated with rapid myeloid proliferation presenting as leukocytosis, although leukocytosis did not necessarily indicate that differentiation syndrome was present [28].

Overall, 34 patients (19.3%) of patients with R/R AML achieved CR with enasidenib, with a slight advantage in *IDH2*-R172 mutations (24.4%) vs. *IDH2*-R140 (17.7%). The objective response rate (ORR) for all R/R AML with *IDH2* mutation was 40.3% with a median duration of 5.8 months and OS of 9.3 months [29]. Similar to CR rates, *IDH2*-R172 achieved higher ORR (53.3%) compared to *IDH2*-R140 (35.4%). Median time to first response was 1.9 months and 87.3% achieved response by cycle five. For patients who attained CR/CRi, median survival improved to 19.7 months [28].

Results of this study helped to clarify an ideal dose of enasidenib, toxicity profile, and establish clinical benefit in R/R AML. On August 1, 2017, enasidenib was granted approval for treatment of adult patients with R/R AML with an *IDH2* mutation [28]. Enasidenib shows a clear benefit in R/R AML for CR, ORR and OS. Additionally, 10% of patients proceeded to transplant indicating that enasidenib may have a role as a bridge to transplant. Importantly, unlike cytarabine-based treatment regimens, failure to show an early response with enasidenib did not foreshadow a treatment failure indicating it might be necessary for patients to receive several cycles to induce a response. Treatment related AEs including hyperbilirubinemia, and differentiation syndrome but infections were extremely low when compared to traditional AML treatments. Since enasidenib appears to induce differentiation and is not myeloablative, it may be very valuable in treating patients with *IDH2* mutations as they are spared the hematological toxicity present with traditional chemotherapy [28].

Further studies should examine the mechanism behind the more favorable response with *IDH2*-R172 mutations. Interestingly, the extent of 2-HG suppression did not correlate with clinical response, and despite a decrease in levels, some patients remained non-responders. This suggests that another pathway may be responsible for enasidenib’s mechanism of action that needs to be explored. Enasidenib continues to be investigated in randomized phase III studies (NCT03839771) as well as maintenance therapy post-transplant (NCT03728335) and in combination with Vyxeos in R/R AML (NCT03825796).

Shortly after the approval of enasidenib, the IDH1 inhibitor ivosidenib was approved on 20 July 2018 for the treatment of R/R AML with *IDH-1* mutations [29]. FDA approval was based off of a multi-center, open-label, single arm, dose-escalation and expansion trial (NCT02074839), with primary efficacy endpoint of rate of CR + CRi. Ivosidenib was administered at 500 mg daily until disease progression, advanced toxicity, or HSCT. At the time of primary efficacy analysis, 125 patients had completed 6 months of therapy, and CR/CRi was achieved in 30% of the primary population (21.6% = CR), with lower response rates seen in patients with adverse cytogenetics, R132H mutation, HSCT, transfusion-dependency, and history of prior therapies. When this was expanded to follow-up of 8.3 months, 174 patients were included in the analysis, and CR/CRi rates improved to 33% of the time (25% = CR) [25,29]. For the subset of patients with CR/CRi, median duration of responses was 8.2 months. However, responses were much more durable for CR (10.1 months) than CRi (3.6 months) [29]. Additionally, 37% of patients went from transfusion dependent to transfusion independent, which is clinically meaningful in this population. The most serious AEs were differentiation syndrome, leukocytosis, tumor lysis syndrome, dyspnea, and Qtc prolongation, although relatively rare. Patients who had a response with ivosidenib overall had fewer infections and episodes of febrile neutropenia than non-responders [25]. While it is unusual for the FDA to grant approval based off of a single arm study, the study provided substantial evidence of efficacy and safety, and the rate of CR/CRi, along with rate of conversion from transfusion dependent to independent, justified its approval. Possibly one of the most significant findings in this study, is that in patients who achieved CR/CRi, 21% of them had no detectable IDH mutations by PCR at the end of the study [25]. This indicates that not only does ivosidenib have value in achieving CR/CRi, but it may also be a curative tool against *IDH1* mutations.

From this study, a subset of 34 patients with newly diagnosed AML were selected for further analysis (nine from dose escalation and 25 in dose expansion phase) with ivosidenib monotherapy at 500 mg daily. Almost one third of patients (30.3%) achieved CR and 42.4% achieved CR/CRi. Median OS was 12.6 months. At one year, 61.5% of CR/CRi and 77.8% CR were still in remission. Interestingly, nine out of 14 patients who achieved CR/CRi cleared the mutation, suggesting that not only does monotherapy with ivosidenib produce durable remissions for newly diagnosed AML, but it may also alter the biology of *IDH1* positive AML itself [3].

Overall, ivosidenib was well tolerated with the most common side effects being diarrhea, nausea, fatigue, anorexia and differentiation syndrome that did not require treatment cessation [3]. Data regarding long term follow-up data and phase III trials still need to be reported, but ivosidenib may prove to be an effective genetic pharmacotherapy for both R/R AML and newly diagnosed AML.

Olutasidenib, a highly potent, orally active, selective inhibitor of IDH1m currently undergoing phase II study, is a recently developed agent in this class with promising potential for drug approval. The phase I study (NCT02719574), performed in patients with *IDH1*m R/R or treatment naïve (TN) AML or myelodysplastic syndromes (MDS) unsuitable for standard therapy, has shown promising results, both as a single agent (SA) olutasidenib and olutasidenib in combination (COMBO) with AZA. In total, 32 patients were treated with SA (26 with AML) and 46 patients (39 with AML) with COMBO. Dosages used included 150 mg once daily (QD) (SA or COMBO) and 300 mg QD (SA) or divided twice daily (BID) (SA or COMBO). Outcomes showed ORR of 41% (CR: 15%) in SA and ORR of 46% (CR: 23%) in COMBO. Median survival rates for SA was 8.7 months and 12.1 months for COMBO in R/R AML patients, and 8.8 months for SA in TN AML (not reached for COMBO in TN AML) [33]. Similar to Ivosidenib, olutasidenib exhibited clearance or significant reduction (variant allele fraction <1%) in *IDH1* mutations in patients with either TN or R/R AM. In fact, this phenomenon was observed in 40% of R/R and TN AML patients (*n* = 10/25) [33], which is of higher proportion when compared to that of Ivosidenib.

Interestingly, there were no dose-limiting toxicities (DLT) observed. Both SA and COMBO cohorts experienced similar AEs, with the most common being hematologic-thrombocytopenia, febrile neutropenia, anemia, and leukocytosis. Differentiation syndrome, QTc prolongation, and transaminitis occurred rarely and resolved with transient hold of olutasidenib. Of note, an impressive 48% of SA and 42% of COMBO treated patients previously transfusion-dependent became transfusion-independent [33]. Overall, olutasidenib has shown positive outcomes on multiple fronts in terms of clinical response, clearance of genetic mutation, and tolerance, and is currently undergoing phase II studies in multiple *IDH1*m AML populations. If results show strong efficacy, this drug may be approved in the near future [33,34].

### 3.3. TP53

Tumor protein p53 (*TP53*) normally acts as a tumor suppressor gene, however it is frequently inactivated by missense mutations in the DNA binding domain that lead to protein unfolding, decreased thermos-stability, and loss of DNA binding and transcription function [35]. *TP53* mutations are found in over 50% of cancers and account for 5–10% of de novo MDS and 25–30% of therapy related MDS [36]. Rates of *TP53* are even higher in patients with a complex karyotype (70%) and is associated with poor prognosis and median survival of only 5.4 months [36]. Current therapeutic agents targeting *TP53* are limited. HMAs are the preferred treatment but only result in CR rates of 20–30% with a median OS of 6–12 months [35]. Patients with MDS or AML with *TP53* mutations are a distinct molecular subgroup that traditionally carry poor prognosis, but new developments including 10-day decitabine, APR-246, and mouse double minute 2 inhibitors (MDM2) stabilizers seem like promising methods to restore appropriate apoptosis in cancer lineages.

During a recent study, Welch et al. sought to identify which molecular markers confer response to decitabine by tracking patters of mutations through enhanced exome and genome sequencing, and correlate changes in mutational burden with clinical response. Patients fell into one of three groups: >60 with AML, R/R AML, or transfusion-dependent MDS. A total of 84 patients with AML or MDS were enrolled in the study. While the study was not specific to TP53 mutations and sequencing was undertaken to detect all common mutations within eight genes (*TP53*, *DNMT3A*, *IDH1*, *IDH2*, *ASXL1*, *SRSF2*, *U2AF1*, and *SF3B1*), the findings of this study showed a robust response and favorable clinical response to a 10-day decitabine regimen in patients with *TP53* mutations [37].

The first 39 patients constituted the discovery cohort. Blast clearance was achieved in 22 patients, including all of the patients with *TP53* mutations. Welch et al. realized the significance of this early on and gathered an additional 60 patients with MDS/AML for data analysis. A total of 14/60 patients had *TP53* mutations and all of them experienced blast clearance with decitabine vs. 17 of 46 patients with wild-type *TP53* (*p* < 0.001). Overall, 21 of 21 patients with *TP53* mutations (100%) versus 32 of 78 patients with wild-type TP53 (41%) achieved CR/CRi or a morphologic leukemia-free state (MLFS) with 10-day decitabine treatment [37].

Post-hoc analyses of cytogenic risk unsurprisingly showed that patients with *TP53* mutations had unfavorable risk cytogenetics (20/21 patients). However, contrary to what one might expect, OS was not significantly affected by high-risk genetics or the presence of the *TP53* mutation. Patients with high-risk genetics had a median OS of 11.6 months, compared to 10 months with favorable or intermediate risk (*p* = 0.29) and patients carrying the *TP53* mutation had a median OS of 12.7 months vs. 15.4 months in wildtype (WT) p53 (*p* = 0.79) [37].

The results of this study were quite shocking, as they were in stark contrast to the poor response rates achieved with standard 7 + 3 chemotherapy. Historically, only 20–30% of patients with *TP53* mutations receiving 7 + 3 had an initial response, and dismal median survival of only 4–6 months, and OS of 10 months after HSCT [37]. It remains unclear whether decitabine truly led to improved outcomes, or if conventional 7 + 3 chemotherapy was causing more detrimental outcomes. Either way, outcomes with decitabine are encouraging and remain a continued avenue of exploration. While results were short-lived with decitabine, the prolonged OS time may provide enough time to be used in a regimen as a bridge to transplant.

This study also illuminated several important caveats about decitabine use. Firstly, responses to decitabine may be slow and patients can require several cycles before mounting a clinical response. Secondly, patients achieved CR/CRi before *TP53* mutational clearance, suggesting that decitabine may induce differentiation prior to eradication of *TP53* mutated leukemic cells. The lack of complete mutational clearance is important to recognize as this may continue to act as a driver mutation and may explain why remission remains short-lived. It might be necessary to continue with decitabine for prolonged periods of time in order to eradicate the possibility of *TP53* resurgence.

Overall, the use of 10-day decitabine showed promising results in one of the most notoriously challenging prognostic groups in AML. The use of decitabine equalized the rate of survival among AML patients with unfavorable-risk cytogenetic profiles, *TP53* mutations, or both, with patients who had an intermediate-risk cytogenetic profile. Although not curative, decitabine may help induce clinical remission for longer periods of time, providing a longer opportunity for a bridge to HSCT, and should continue to be explored in prospective trials in combination with other emerging drugs targeting *TP53* mutations. Decitabine is currently being investigated for AML and high-risk MDS along with midostaurin (NCT04097470).

At the 2020 European Hematology Association Congress annual meeting, promising data were presented from two ongoing phase II clinical trials of APR-246, a novel TP53-activating drug. The mutant TP53 reactivating compound APR-246, also known as eprenetapopt, (PRIMA-1Met) is a small molecule that specifically targets the TP53 core domain, promoting refolding of proteins, and restores TP53 to its wild type functionality, allowing apoptosis to occur [38]. In a small, multicenter, phase Ib/II, dose-escalation trial of APR-246 and AZA in adult patients with *TP53* mutant MDS or oligoblastic AML (≤30% blasts), ORR by International Working Group (IWG) 2006 criteria was 100%, with nine patients achieving CR and two achieving CR based on bone marrow (NCT03072043). All CR patients had high *TP53* at baseline which normalized with serial assessment. Deeper genomic analyses showed transcriptional activation of TP53 and pathways involved in cell cycle arrest, apoptosis, and DNA repair, and while not yet significant, the combination of APR-246 and AZA had a trend towards improved OS [36].

While it has not yet received FDA approval for standard use, it has received orphan drug and fast track designations from the FDA to be used in *TP53* positive MDS as of 16 April 2019, and breakthrough therapy designation as of 30 January 2020. Studies are currently underway examining APR-246 in *TP53* mutant MDS, AML, as well as chronic lymphocytic leukemia (CLL) and mantle cell lymphoma (MCL) and are in different phases of study. A phase III clinical trial of APR-246, plus AZA for MDS is currently enrolling patients (NCT03745716), as well as a phase II study (NCT03931291) investigating APR-246 as maintenance therapy post HSCT in MDS and AML [39]. If successful, the addition of APR-246 to HMA for the treatment of *TP53* will bring about much needed change in a grim disease.

Another therapeutic agent being investigated in *TP53* mutant AML are Mouse double minute 2 (MDM2) inhibitors (MDM2-i). MDM2 is a negative regulator of TP53 and therefore MDM2-inhibitors help reactive wild-type TP53. Drugs called nutlins were the first small molecule inhibitors. RG7388 (idasanutlin) is a second-generation nutlin. In a multicenter phase I/Ib study, idasanutlin was evaluated in AML patients as monotherapy or in combination with ara-C. The monotherapy extension arm included patients >70 years or >60 years with comorbidities. The combination extension arm included R/R patients who had not undergone more than two prior regimens or HSCT. Unfortunately, enrollment into the monotherapy arm was prematurely discontinued for prolonged myelosuppression and early death. The combination arm proved to be more promising, with a CR rate of 25%, with almost one quarter of patients sustaining remission at one-year follow-up. MDM2 protein expression was evaluated by intracellular flow cytometry on peripheral blood leukemic blasts and stem cells, with higher expression of MDM2 leading to a higher likelihood of achieving CR, indicating that MDM2 expression could potentially be used as a biomarker of response with these particular agents [40]. Given the efficacy in phase I/Ib AML studies, idasanutlin is currently undergoing evaluation in a phase III trial in combination with cytarabine vs. cytarabine alone for R/R AML patients (NCT02545283). Idasanutlin is also being explored for R/R AML in combination with other apoptotic agents such as the BCL2 inhibitor venetoclax. (NCT02670044) [40].

Various additional small molecule MDM2-inhibitors are currently being investigated. A Phase I trial in R/R AML with MK-8242 showed poor efficacy with only 1/24 patients responding. A phase Ib study examining AMG232 with or without trametinib (*MEK* inhibitor) again showed no benefit on the monotherapy arm, and only one CR in combined therapy arm. AMG-232 is currently being evaluated with decitabine in newly diagnosed and R/R AML (NCT03041688). Similarly, the compound HDM201 which inhibits HDM2-p53 interaction is being investigated in WT-p53 which promising preliminary results of 20.6% CR/CRi and partial response. Currently, HDM201 in combination with chemotherapy in newly diagnosed and R/R AML is underway (NCT03760445). Other drugs under investigation include DS-3032b (milademetan) in combination with AZA (NCT02319369), and in combination with cytarabine and quizartinib (NCT03552029) [40].

Mouse double minute X (MDMX) is similar to MDM2 in that it represses *TP53* transcription. MDMX- inhibition is thus being examined in combination with MDM2-i to assess for synergistic effects. ALRN-6924 is a peptide that has been structurally stabilized in an α-helical configuration to mimic the inhibitor binding region of TP53. This configuration allows for the binding of both MDM2 and MDMX and leading to indirect inhibition of both. A phase I/Ib study was recently completed evaluating ALRN-6924 alone and in combination with cytarabine in R/R AML or advanced MDS patients with *TP53* mutation. While no drug-limiting toxicities were identified, preliminary results showed only modest clinical improvement at best [40].

*TP53* mutated AML remains challenging to treat, and thus various combinations of therapeutic agents are in development and under investigation in various combinations with the hope of improving remission rates in this unfortunate cohort.

## 4. Non-Genetically Targeted Therapies

### 4.1. Vyxeos

In 2017, liposomal daunorubicin and cytarabine (Vyxeos) was approved for patients with newly diagnosed t-AML or AML-MRC [41]. Both daunorubicin and cytarabine exert their cytotoxic effects at the level of DNA synthesis and repair, synergistically killing leukemic cells both in vitro and in vivo in murine models [42,43]. Different pharmacokinetic and pharmacodynamic profiles of the two drugs make it difficult to preserve a particular ratio in the blood, and thus Vyxeos, a liposomal carrier with a fixed 5:1 ratio of cytarabine to daunorubicin was developed [44]. Vyxeos maintains the fixed ratio for up to 24 h, which is longer than traditional 7 + 3, and results in greater drug exposure and uptake by tumor cells. Additionally, the rigid bilayer of the liposome reduces the rate of extravasation thus prolonging exposure to the tumor [45]. Approval of Vyxeos was based on an open-label phase III randomized control trial (RCT) comparing Vyxeos to 7 + 3 (NCT01696084) in patients 60–75 with newly diagnosed t-AML or AML-MRC [46]. Compared to conventional 7 + 3, Vyxeos displayed better median OS (9.6 versus 6.0 months; HR 0.69; 95% CI 0.52 to 0.90) as well as a higher remission rate (48% versus 33%) while maintaining a similar safety profile. Despite a variation in baseline age, race, Eastern Cooperative Oncology Group (ECOG) performance status, and proportion of unfavorable cytogenetics, the treatment arm remained as an independent predictor for OS [46]. Presently, multiple clinical trials are seeking to expand the clinical applicability of Vyxeos to R/R AML, and it is being studied in combination with Enasidenib (NCT03825796), Quizartinib (NCT04209725) or venetoclax (NCT03629171).

### 4.2. Venetoclax

BCL2 is an anti-apoptotic protein overexpressed in multiple lymphoid and myeloid malignancies, including AML cells, especially Leukemia Stem Cells (LSC) which may be dependent on BCL2 for survival. Venetoclax, a BCL2-inhibitor showed modest results as a single agent in R/R AML with 19% response rate, but more importantly showed synergistic effects with LDAC and HMA in a multicenter phase I/II trial. CR/CRi was achieved 54% of the time with venetoclax plus LDAC and 67% with HMA, with median OS of 10.4 months and 17.5 months, respectively [6]. While these trials were non-randomized, it led to the accelerated approval of venetoclax in combination with azacitidine or decitabine or low-dose cytarabine in November 2018 for newly diagnosed patients over the age of 75 who were ineligible for standard induction chemotherapy. It was shown that most patients benefited from venetoclax within one cycle. AEs of venetoclax included prolonged myelosuppression and grade 3/4 cytopenias, infections, tumor lysis syndrome, mild gastrointestinal symptoms and significant interaction with CYP3A4 inhibitors warranting dose reduction.

Recently, results of VIALE-A, a confirmatory trial for evaluation of venetoclax in combination with azacitidine in patients with previously untreated AML, was officially published. Of note, the FDA had granted accelerated approval back in 2018 as stated above. VIALE-A was a phase III randomized, double-blind placebo-controlled study (NCT02993523) that randomized 431 treatment-naïve patients with AML, ineligible for intensive induction therapy or age ≥75, to 2:1 AZA (75 mg/m^2^ on days 1 to 7) plus venetoclax (400 mg daily) or AZA + placebo [47]. The median overall survival was 14.7 months in treatment group compared to 9.6 months in control group. CR/CRi rates were 66.4% vs. 28.3 (*p* < 0.001) in treatment vs. placebo groups. Especially exciting, is its composite CR rates in patients with poor cytogenetics, including 55% in patients with *TP53* mutations, approximately 75.4% in patients with *IDH1/2* mutations, which is significant when compared to CR rates of 0–36.4% in patients with the same mutations treated with AZA alone. AEs consisted mainly of nausea, and hematologic abnormalities—particularly thrombocytopenia and neutropenia [47]. VIALE-C is a double-blind, placebo-controlled phase III study (NCT03069352), where 211 patients with previously untreated AML unsuitable for intensive chemotherapy or ≥75 years were randomized 2:1 venetoclax plus LDAC or placebo plus LDAC. Median OS was 7.2 vs. 4.1 months in treatment vs. placebo, with 25% reduction in risk of death, although not statistically significant. CR/CRi rates were 48% versus 13% for venetoclax plus LDAC and LDAC alone. In patients with poor cytogenetic risk, patients with *IDH* mutations treated with Venetoclax plus LDAC had a CR/CRi rate of 57% compared to CR/CRh rate of 42% in AML patients treated with ivosidenib (IDH1 inhibitor) [48,49]. Overall, both studies show clinically meaningful improvement and are contributory to the gaining popularity and interest in combination therapies.

Triplet therapy, combining a minimally myelosuppressive third agent with a venetoclax and AZA backbone, seems to be promising, with various combinations currently being studied in ongoing trials. While azacitidine combined with venetoclax have been efficacious in both newly diagnosed and relapsed/refractory (R/R) AML, potential drug resistance is always of concern. The most recognized mechanism of resistance to venetoclax is overexpression of MCL1, another anti-apoptotic protein which can predominate when BCL2 is inhibited. Thus, MCL1-inhibitors are being studied in combination with BCL2-inhibitors, and indirect targets within the MAPK pathway, TP53 and CDK9 pathways [50].

Of particular note, pevonedistat (PEV, TAK-924/MLN4924), a novel inhibitor of NEDD8-activating enzyme (NAE), is currently being investigated in combination with venetoclax + AZA for its possible enhancement of therapeutic efficacy in overcoming malignant apoptotic resistance. Mechanistically, NEDD8 is a ubiquitin-like protein that is activated by an E1 enzyme (NAE), which is then transferred to E2 then to downstream substrates such as cullin-RING ligases (CRL), CRL-NEDD8 complex then act as ubiquitin ligases that target substrates for proteasome degradation. NAE inhibition theoretically leads to CRL inactivation and subsequent CRL substrate accumulation induces cell cycle arrest and death. PEV specifically induces NOXA, a pro-apoptotic protein, that can neutralize MCL-1 inducing apoptosis [51,52]. In the phase Ib study of PEV plus azacitidine doublet therapy in patients with relapsed/refractory AML and MDS unsuitable for high-dose induction therapy, treatment was well tolerated in the older population with known AE of AZA and transaminitis as AE due to PEV, but a nonoverlapping toxicity profile. There was an 83% ORR in patients who received at least six cycles of therapy. In *TP53* mutant AML patients, the CR/PR rate was 80%, with responses seen in patients with refractory disease [52]. The phase III trial is currently ongoing with this doublet as frontline treatment for patients with higher-risk myelodysplastic syndromes (HR MDS), chronic myelomonocytic leukemia (CMML), and low-blast AML and results will be published in the near future [53]. Of note, prior preclinical studies indicated synergistic effects of PEV and venetoclax against AML cell lines [53]. Given these results, triplet therapy with PEV, AZA, and Venetoclax (PAVE) is now currently undergoing phase Ib trials, using a varied 3+3 design. In addition, correlation of pretreatment levels of BCL2, BCLXL, MCL1, BAX or BAK with response to treatment, as well changes in NOXA expression, will also be evaluated, to assess efficacy in cytogenetically poor patients [54].

Another triplet therapy undergoing phase Ib/II study is IDH1 inhibitor ivosidenib with venetoclax with or without AZA (NCT03471260). Here, patients age ≥ 18 with *IDH1* mutated high-risk MDS or AML are enrolled in one of three cohorts (cohort one: ivosidenib plus venetoclax 400 mg, cohort two: ivosidenib plus venetoclax 800 mg, and cohort three: ivosidenib plus venetoclax 400 mg and AZA. So far, composite CR was 78% overall, and 67%, 100%, and 67% in each respective cohort. If data continue to be promising, this may have potential to become standard line of therapy in AML patients with *IDH1* mutation [55].

APR-246 targets mutant p53 through its conversion to reactive electrophile methylene quinuclidinone (MQ) and binding the p53 core domain, thereby restoring p53 tumor suppressing, proapoptotic and cell arrest functions [35]. As described earlier, APR-246 combined with azacitidine in patients high-risk AML with *TP53* mutation is currently undergoing phase III trials. However, APR-246 as a triple therapy, coupled with venetoclax and azacitidine, is also undergoing phase I studies (NCT04214860) in patients with *TP53* mutation containing AML that is either untreated or have been through prior therapies with azacitidine or decitabine [56,57].

Additional studies for patients with untreated AML include AZA and venetoclax in combination with Gemtuzumab-oligomycin (GO, also known as CMA-676) (NCT04070768) and avelumab (PD-L1 inhibitors—NCT03390296). In R/R AML, venetoclax is being studied in combination with CDK9-inhibitors (NCT03441555), JAK2 inhibitor (NCT03874052), FLT3 inhibitors such as quizartinib (NCT03735875, NCT03661307) or gilteritinib (NCT04140487, NCT03625505), MCL1 inhibitor (NCT03672695), MEK/MDM2 inhibitor (NCT02670044), XPO1 inhibitor (NCT03955783) and anti-CD33 monoclonal antibodies (NCT03867682) [6].

### 4.3. Glasdegib

Another targetable pathway includes the Hedgehog (Hh) signaling pathway, which normally regulates cell differentiation and renewal during embryonic development, but has been found to be abnormally upregulated in leukemia and leukemia stem cells (LSC) [5]. The transmembrane protein smoothened (SMO) is essential for Hh activation and is what allows LSC to remain dormant and thus resistant to chemotherapy. Glasdegib, a SMO inhibitor, inhibits the Hh signal and causes LSC to re-enter the cell cycle, allowing them to be susceptible to chemotherapy. Glasdegib has demonstrated clinical activity in both phase I and Ib/II trials in untreated AML and MDS in conjunction with 7 + 3. For adults with untreated AML who were unfit for chemotherapy, the combination of glasdegib plus LDAC improved OS from 4.3 months to 8.3 months compared with LDAC alone, and also increased rates of CR/CRi and MLFS [5]. This prompted a phase III trial examining the efficacy of glasdegib with other chemotherapy regimens. The BRIGHT AML 1019 trial (NCT03416179) is an ongoing study composed of two independent phase III, randomized, double-blind trials that evaluate intensive chemotherapy with 7 + 3 plus glasdegib vs. placebo, and low intensive therapy with AZA and glasdegib vs. placebo. Hopefully the results of this study will lead to approved combinations of glasdegib with 7 + 3 and AZA.

### 4.4. Oral Azacitidine

Azaciditine, a hypomethylating agent, is a nucleoside analog of cytidine that halts protein synthesis through its incorporation into DNA, blocking cytosine methylation by irreversible, noncompetitive inhibition of DNA methyltransferase (DNMTs). Subsequent cell division in absence of DNMTs causes progressive DNA hypomethylation, reactivating genes, including tumor-suppressor genes, that were previously silenced through malignant hypermethylation. This medication has traditionally been administrated intravenously either as monotherapy or in conjunction with other cytotoxic agents as induction chemotherapy and its role as maintenance treatment has only been recently described. In one study, patients with MDS, chronic myelomonocytic leukemia (CMML), and AML who received subcutaneous azacitidine as maintenance therapy showed good tolerance with a median OS of 20 months in treatment patients versus 8.2 months for the entire population [58]. A newer study performed in older patients with AML in CR/CRi given subcutaneous azacitidine exhibited significant improvement in DFS, with 12-month DFS at approximate 64% in the azacitidine cohort and 42% for control subjects [58,59].

Oral azacitidine (CC-486) was recently approved by the FDA on September 1, 2020 for maintenance therapy in adult patients with AML who have achieved first CR or Cri following intensive induction chemotherapy unable to complete aggressive curative therapy. The phase I trial, initially performed in patients with MDS, CMML, and AML (limited to patients not tolerant of standard curative measures), showed encouraging results with 38% of AML patients achieving an overall response in part one (patients receive one cycle of subcutaneous azacitidine at 75 mg/m^2^ for 7 of 28 days, followed by CC-486 at 120–600 mg daily for 7 days in repeating 28-day treatment cycles) and 47% of AML patients achieving an overall response in part two (extending dose regimes of CC-486 at 300 mg daily or 200 mg twice daily for 14 or 21 days of 28-day cycles). The drug was generally well tolerated with an maximum tolerated dose (MTD) of 450 mg, limited by development of severe diarrhea, and no signs of drug accumulation [60,61]. Most common grade three/four AEs were gastrointestinal disturbances, including diarrhea, nausea, vomiting, but also including febrile neutropenia, pneumonia, and syncope [61]. Surprisingly, a reduction in DNA methylation levels in oral azacitidine was comparable to those of subcutaneous azacitidine, in fact, extending CC-486 dosing schedules prolonged methylation reversal [61].

Due to these positive results with the promise of administration convenience as an oral agent, a phase III trial (NCT01757535), called QUAZAR AML-001 Maintenance trial (CC-486-AML-001), a randomized double-blind, placebo controlled study, was initiated to test for efficacy and safety of CC-486 as maintenance therapy specifically in older patients, age ≥55 years, with AML in first CR/CRi after induction chemotherapy with or without consolidation chemotherapy, who were not candidates for HSCT [62,63]. A total of 472 patients either received oral azacitidine at 300 mg or placebo once daily. The group treated with azacitidine had statistically significant increase in overall survival, 24.7 months in treatment arm versus 14.8 months in placebo arm, with consistent OS benefit in patients with either CR or Cri [63]. Median RFS was also significantly prolonged with CC-486 (10.2 months vs. 4.8 months). Benefits were witnessed through all subgroups, including patients with poor cytogenic-risk disease, MRD, or were ≥65 years old. Most frequent AEs were again grade one or two gastrointestinal events, with most common being nausea, vomiting, and now diarrhea (did not reach dose-limiting toxicity for severe diarrhea). Most common hematologic AEs were neutropenia, thrombocytopenia, and anemia. Because the drug was not intravenous, infections were relatively rare [63,64].

Overall, oral azacitidine is a drug with many advantages to patients, including ease of administration, reduced healthcare cost implications, and allowance for extended administration. CC-486 not only exhibited clinically meaningful improvements, the relatively benign and manageable safety profile makes it especially exciting and a candidate for integral treatment for older adults with AML in remission. CC-486, Onureg, has since been approved by the FDA.

### 4.5. Antibody–Drug Conjugates

Antibody–drug conjugates (ADCs) are another line of promising immunotherapies in the battle against AML. The CD33 antigen (SIGLEC-3) is highly expressed on leukemic myeloblasts, approximately 90% of AML myeloblasts, and has been a popular target for immunoconjugate drugs. gemtuzumab ozogamicin (GO), a humanized anti-CD33 monoclonal antibody conjugated to the cytotoxic agent calicheamicin [65]. Mechanistically, GO binds to the CD33 antigen leading to its internalization and release, intercalating DNA and inducing double-stranded DNA breaks with subsequent cellular death [66]. GO is a promising drug with a complicated history. It was initially approved at 9 mg/m^2^ as an addition to induction chemotherapy for older patients with R/R AML not suitable for intensive therapy [67]. Phase II trials were encouraging, indicating an overall response rate of 34% in patients below 60 years old and 26% in patient over 60 years old, with significantly lower rates of complications as well as tolerability without hospitalization [67]. This prompted accelerated FDA approval for treatment of patients over 60 years of age with AML in first relapse who may not tolerate aggressive chemotherapy [68]. The Southwest Oncology Group (SWOG) led further investigation to complete a phase III RCT comparing the addition of 6 mg/m^2^ GO (dose previously shown to saturate CD33 receptors) to induction and consolidation chemotherapy vs. standard therapy in patients under 60 with newly diagnosed AML. However, results were poor, as study failed to show any improvement in DFS, OS or CR [68]. Similar trials by the French GOELAMS group, and French ALFA group also failed to show improvement in remission, despite some reduction in relapse rate. Ultimately the SWOG-S0106 trial was terminated early due to increased deaths in induction, concern for veno-occlusive disease (VOD) and lack of improvement in relapse rate, DFS, and OS. GO was removed from the market in 2010 [69].

After a period with a paucity of new drug regimens, GO was re-examined at lower doses, and further studies demonstrated that a dose of 3 mg/m^2^ was safe and effective, motivating the FDA to re-approve GO for newly diagnosed and R/R CD33+ AML [66,70]. A meta-analysis analyzing GO as a single agent in R/R AML showed that GO could be safely administered at a fractioned dosing regimen of 3 mg/m^2^ on days 1, 4, and 7 with a more favorable risk profile [66]. Efficacy in R/R CD33+ was supported by the MyloFrance one trial, a single arm study examining fractionated dosing of GO that showed 26% CR with a median RFS of 11.6 months. On 2 September 2017 the FDA re-approved GO at the fractioned dosing for adults with R/R AML, which was then extended to pediatric patients above the age of two [66].

Similar studies were performed with GO in newly diagnosed CD33+ AML. ALFA-0701, a randomized phase III trial of 7 + 3 for induction and consolidation chemotherapy, with or without GO, found median EFS of 17.3 months with GO as compared to 9.5 months in the control arm, although there was no difference in OS. Early mortality remained slightly higher in the GO arm, with high attribution to VOD and hemorrhage, although much reduced from the higher doses of GO [71]. It is important to note that though there seems to be a positive correlation between GO Cmax and probability of developing VOD, there is no relationship between Cmax and complete remission. Furthermore, these EFS results were comparable in various subgroups, with the exception of the subgroup with adverse cytogenic risk [71]. In practice, GO is often considered as additive therapy to 7 + 3 induction for a curative goal in patients with favorable cytogenic risk rather than in patients with adverse prognosis undergoing hematopoietic cell transplantation. Overall, the benefit of using GO in this population would outweigh the risk of patients developing VOD.

GO was also examined as a monotherapy in AML19, a phase II/III trial in elderly individuals with newly diagnosed AML with poor performance status who were unfit for chemotherapy. Treatment with GO consisted of induction with 6 mg/m^2^ GO on day 1 and 3 mg/m^2^ GO on day 8, followed by maintenance therapy with 2 mg/m^2^ every four weeks. The control arm received best supportive care. While there was a significant increase in OS with GO, the effect was modest at 1.3 months. Additionally, there was no benefit for patients with CD33 expression less than 20% or those with adverse cytogenetics. Although GO monotherapy is not curative, it may be beneficial in patients who are unfit for standard chemotherapy and desire even a short survival benefit [71]. GO with 7 + 3 has shown statistically significant survival advantage in patients with FLT3 mutant AML (NCT03900949), providing another avenue of opportunity for studies examining this regimen in combination with FLT3-inhibitors [13].

One of the issues facing GO, is that it remains unclear which patient population it will benefit the most, and how to integrate it best into practice. FDA approved GO without setting a threshold for CD33 level expression, or specifying which cytogenetics respond the best. The literature reveals conflicting results about the effect of CD33 expression and genotype. In a pediatric study of 0–29 year-olds, GO was found most effective in patients with favorable cytogenetics and higher expression of CD33, showing significant improvement in CR rates, relapse rate, EFS and DFS, although no improvement in OS [72]. Other studies found a positive association between GO plus chemotherapy and the genotype at a single nucleotide polymorphism (SNP) at a splice site of the CD33 gene which affects the expression of the extracellular epitope recognized by GO [73]. A change from the common C allele to the T allele truncates the IgV domain recognized by GO, which could potentially lead to decreased efficacy of GO. However, these results were not replicated in adult populations [65,74]. Further research is still needed to decipher the best biomarkers to predict response for CD33 positive AML.

## 5. Immunotherapies

### 5.1. Immunotherapy and AML

Parallel to novel targeted therapy, immunotherapy is a promising approach to AML treatment, particularly in transplant-ineligible patients and those with R/R disease. From immune checkpoint inhibitors to disease-specific vaccines, multiple immune modulating strategies are being developed with variable degrees of success in preclinical and clinical models.

### 5.2. Immune Checkpoint Blockade

Immune checkpoint inhibitors have shown favorable outcomes in multiple solid malignancies, however, were less successful when applied to patients with AML. PD-1 overexpression is found in 40% of patients with AML, which may be contributory to immune-evasion and AML cell growth [75]. Several trials explored the efficacy of immune checkpoint inhibitors when combined with other agents. Induction regimens with nivolumab, cytarabine and idarubicin was tested in a phase I/II single-arm study with 44 patients with newly diagnosed AML or high-risk MDS [76]. Nivolumab was administered on day 24 of chemotherapy and continued every 2 weeks for up to a year in responders. The study group observed a CR/CRi rate of 77% (*n* = 34/44), 53% of which were MRD negative by flow cytometry. Grade three and four immune-related adverse events were observed in seven cases and involved skin (rash) and gastrointestinal tract (transaminitis, pancreatitis and cholecystitis).

Immune checkpoint blockade was also tested in older and less fit patients. It had been observed previously that older adults with poor response to HMAs have PD-1 overexpression, and thus it was hypothesized that nivolumab may increase sensitivity of leukemoid cells to HMA [75]. The SWOG 1612 trial is a phase II/III trial assessing the survival benefits of two experimental arms: AZA plus nivolumab, and AZA plus midostaurin. However, due to early signals for excessive grade five events, the study group temporally suspended the trial [77].

Notably, in the 61st American Society of Hematology (ASH) annual meeting, Chandhok et al. presented the study design of a multicenter, single-arm phase II trial on induction combination ivosidenib and nivolumab [78]. The primary endpoint is to assess safety and tolerability of this regimen in patients with MDS or R/R AML with mutated IDH1 (NCT04044209). Enrolled subjects will have the option to proceed to transplant if eligible. This trial will provide data on feasibility of an induction regimen different to the conventional chemotherapy.

Checkpoint inhibitors are also being evaluated as maintenance therapy and in R/R AML. A maintenance regimen with nivolumab yielded promising results in a single-arm, phase II study with high-risk AML patients [79]. Preliminary results in 14 patients showed 6- and a 12-month CR duration of 79% and 71%, respectively. The trial is in active recruitment and is expected to complete in October 2021 (NCT02532231). A Phase II trial with ivosidenib in combination with nivolumab in IDH1 mutated R/R AML is also under way (NCT04044209).

### 5.3. CD47—Magrolimab

CD47, or integrin-associated protein, is a transmembrane protein that functions as an anti-phagocytic “do not eat me” signal. In tumor cells, overexpressed CD47 binds signal regulatory protein alpha (SIRPα) on macrophages, resulting in inhibition of myosin accumulation, halting the subsequent phagocytosis [80,81]. Blockade of the CD47 checkpoint may lead to an adaptive anti-tumor response by increasing tumor cell phagocytosis. CD47 upregulation may be found in a myriad of malignancies and was identified as a marker for leukemia stem cells in AML [82,83].

Magrolimab, previously known as 5F9, was the first humanized antibody with anti-CD47 activity to enter clinical development. Phase I studies with elderly patients with AML and MDS examining magrolimab as a monotherapy and in combination with AZA have shown a favorable safety profile, with treatment-related anemias noted as the most common adverse event [84,85]. Notably, preliminary data from ongoing phase Ib trials showed associated ORR of 69% in the magrolimab arm, and 50% CR/CRi rate (NCT03248479) [84]. Interestingly, this study observed that magrolimab plus AZA demonstrated early efficacy in AML patients with mutated TP53. Patients with this mutation often have poor response to conventional chemotherapy because the basis of many chemotherapeutic agents is to inflict enough DNA damage to induce apoptosis. Patients with this mutation, regardless of their cytogenic profile, carry poor prognosis with OS of 7–8 months with treatment, with only 20% of patients who have true CR in response to AZA [86]. In patients that received magrolimab plus AZA, CR/CRi rate in the TP53 mutant AML patients receiving treatment was 78% overall, with a pronounced reduction in TP53 mutant variant allele frequencies, spotlighting Magrolimab as a candidate for frontline therapy in cytogenetically unfavorable AML patients with T53 mutants.

These initial data represented proof-of-concept of efficacy of CD47/SIRPα pathway blockade in AML patients. Unfortunately, studies with other anti-CD47 antibodies (e.g., CC-90002, Celgene, Summit, NJ, USA), were terminated early. Additional CD47 blockading strategies are under development, including anti-SIRPα antibodies (CC-95251, Celgene/NCT03783403) and wildtype SIRPα fusion proteins (TTI-621 and TTI-622, Trillium Therapeutics, Mississauga, ON, USA; NCT02663518, NCT03530683).

### 5.4. CD123—SL-401/Tagraxofusp

Interleukin-3 receptor alpha chain (IL-3Rα), also known as CD123, is another novel therapeutic target in AML treatment. CD123 is overexpressed in multiple hematological malignancies including AML and LSCs [87]. Tagraxofusp or SL401 (Stemline Therapeutics, New York, NY, USA) is an immunotoxin composed of a recombinant protein made of diphtheria toxin fused to human IL-3. Although initially approved by the FDA for adult and pediatric blastic plasmacytoid dendritic cell neoplasm (BPDCN), in December 2018 this agent showed promising cytotoxic activity against CD123-positive AML blast cells in animal models [88]. Clinical data on tagraxofusp remain limited. A phase I/II clinical trial (NCT02270463) is currently assessing the safety and tolerability of tagraxofusp in AML patients who are at risk of relapse and are non-eligible for transplant. Its preliminary results were presented in the 2017 ASH meeting and suggested an acceptable safety profile, with pending efficacy data [89].

### 5.5. Bispecific T Cell Engager

Bispecific T cell engagers (BiTE) are small molecules engineered to facilitate tumor cell death via MCH-independent T cell activation. Structurally, they are composed of two single-chain variable fragments (scFvs): one domain specific to a selected tumor-associated antigen, and the other binds the invariable CD3 domain of the T cell. Due to its small molecular size, BiTE approximates T cell and tumor cells upon engagement, which results in a transient formation of a cytolytic synapse and subsequent tumor cell death via direct release of granzymes and perforins [90].

Given the ubiquitous expression of CD123 in AML cells, CD123-CD3 BiTE products are being explored. Ravandi et al. presented interim results from a first-in-human phase I trial exploring safety and tolerability of XmAb14045 (NCT02730312). The preliminary results included 64 patients, 63 of which were heavily pretreated R/R AML [91]. Cytokine release syndrome was the most common adverse events, although it was generally manageable with premedication and standard supportive care. Notably, weekly dosing of XmAb14045 was associated with anti-leukemic activity with a CR/CRi rate of 23%. The trial is expected to complete in February 2021 and will include patients with AML, chronic myeloid leukemia (CML) with blast crisis, ALL and BPDCN [92].

### 5.6. CAR T Cell Therapy

Chimeric antigen receptor (CAR) T cell therapy gained increasing attention as a novel, potent, treatment approach to many malignancies. This living therapy consists of autologous or allogenic T cells genetically engineered to express a CAR capable of recognizing a preselected tumor antigen, thus achieving selected tumor necroptosis [92].

Multiple antigenic targets were explored in preclinical settings. Myburgh et al. utilized antigen c-Kit (CD117)-targeting CAR T cells and observed elimination of 90% of CD117 positive leukemia cell lines within 24 h in co-culture assays [93]. Notably, Sommer et al. generated CAR T cells targeting soluble FLT3 scFvs, which demonstrated dose-dependent expansion in vivo and cytotoxic activity in murine model-based study [94].

Currently, clinical data of CAR T cells are limited, yet promising. CYAD-01, a CAR T cell product based on the receptor of natural killer group 2D (NKG2D), was administered to patients enrolled in the THINK trial (NCT03018405) [95]. In the hematological arm of this open-label phase I trial, ORR was observed in three out of seven (42%) response-evaluable patients with R/R AML; one patient experienced CR with partial hematologic recovery, and two with CR with incomplete marrow recovery. G3/G4 adverse effects were common and found in five of twelve subjects and included lymphopenia, thrombocytopenia, pneumonitis and cytokine release syndrome [95]. Another ongoing Chinese phase I study observed remission in two patients with multiple relapsed AML treated with a novel compound CAR T cell (cCAR) (NCT03795779) [96]. This cCAR product has two distinct functional CAR molecules, CD33 and CLL1. Rationales for dual targeting include a theoretical superior ablative effect and lower likelihood for relapse secondary to antigen loss in AML cells. This trial is in active recruitment and is expected to complete in September 2020.

Indeed, CAR T remains a promising therapeutic option given its effectiveness and great versatility as it may be designed to target any antigen expressed on the surface of a cell. Nonetheless, challenge arises with AML treatment due to lack of a specific target. For instance, CD117 expression is found on 90% of samples from AML patients, but it is also found physiologically on hematopoietic stem cells, hence, it is designed to be followed by CAR T cell depletion and stem cell transplantation [94]. Similarly, patients from the study reported by Liu et al. all experienced myelo-depletion and required transplantation [96].

### 5.7. Vaccines

CAR T cell therapy is designed to deliver T cells to patients with direct cytotoxic effects. Conversely, cancer vaccines are developed to stimulate durable, systemic immunological memory by administering dendritic cells (DCs). Through prior in vitro protein-based vaccination, these DCs gain the ability to process and present tumor antigens to native T cells, which in turn exert the role of tumor cytolysis [92]. Analogous to CAR T cell therapy, antigen selection is crucial in development of cancer vaccine. Wilms’ tumor protein 1 (WT1) is physiologically found at low levels in gonads, kidneys and normal hematopoietic tissue and is highly expressed in hematological malignancies including AML [97]. Yamaguchi et al. evaluated the safety and efficacy of OCV-501, a synthetic peptide containing natural sequence of WT1, in a randomized, multicenter, double-blind, placebo-controlled trial [98]. Eligible AML patients were 60 years or older, who had achieved CR within two courses of induction therapy and had completed at least one round of consolidation. OCV-501 was given as maintenance therapy once weekly for up to 8 weeks, then followed by biweekly dosing for up to 2 years. 133 patients were enrolled, of which 68 received vaccine. The primary endpoint was DFS, which was numerical higher in vaccine group (12.1 months versus 8.4 months in placebo group) although the difference was not statistically significant. Similar non-significant results were observed in OS: 38.5 months in the vaccine arm vs. 31 months in the placebo arm. Notably, OCV-501 displayed a favorable safety profile during prolonged treatment course: most patients developed mild adverse events (G1 or G2) and the most frequently reported event was injection site induration. Similar results were observed in another phase II trial with a multivalent WT1 peptide vaccine and AML patients in first remission [99]. Reported median DFS from first remission was 16.9 months, with OS not reached but estimated to be ≥67.6 months. Nonetheless, relapse rate and mortality were high and were 68% (*n* = 15) and 46% (*n* = 10), respectively [100].

In a post hoc analysis from a phase I trial, Janssen et al. observed that DCP-001, an allogeneic leukemia-derived dendritic cell vaccine, may prolong length of remission when used as maintenance therapy in intermediate- and high-risk AML or MDS [99]. The ADVANCE II trial is an ongoing multicenter phase II trial (NCT03697707) that is evaluating the effect of DCP-001 on AML patients who are in remission but have persistent detectable MRD. Expected completion is around December 2020.

Novel combination therapies with vaccines are also being explored in preclinical models. These regimens involve vaccine +/− immune checkpoint inhibitor cocktail +/− HMA [101,102]. Although preliminary results are promising, clinical data on human models are currently lacking.

## 6. Drugs in the Pipeline

The treatment of AML is experiencing unprecedented change, with new pharmacotherapies being developed and undergoing trials constantly. New advances and molecular targets of action are in the pipeline, which hopefully, will produce even more options than are currently available, enhancing precision medicine, propelling the field forward and bringing meaningful change to patient outcomes.

Translocations of the mixed lineage leukemia 1 (*MLL1*, *MLL* or *KMT2A*) gene are found in approximately 5–10% of AML, resulting in unfavorable disease. Fusion with other proteins leads to enhanced proliferation without cell differentiation, thus driving the development of leukemia [103]. In pre-clinical trials, the menin-inhibitor MI-3454 profoundly inhibited cell proliferation while enhancing differentiation in AML patients with either *MLL* or *NPM1* mutations. Prior studies with earlier menin-inhibitors demonstrated similar growth arrest and promotion of cell differentiation in patients with *NPM1*- mutations. In November of 2019, the AUGMENT-101 trial, a phase I/II trial evaluating a new highly selective Menin-inhibitor SNDX-5613, began evaluating patients with R/R AML (NCT04065399) [104]. Hopefully SNDX-5613 will show promising results for inhibiting both *MLL* and *NPM1*.

*NPM1* mutations are found in 30% of patients with AML. *NPM1* is a chaperone protein involved in epigenetic regulation through protein shuffling between the nucleus and the cytoplasm. In *NPM1*+ AML, the mutant protein NPM1c is abnormally located in the cytoplasm. Preclinical studies showed that when this protein was relocated to the nucleus by blocking nuclear export with exportin-1 (XPO1) inhibitors, or degraded with proteolysis targeting chimera (PROTAC) therapy, HOX genes were downregulated and cells were able to differentiate, prolonging the survival in an NMP1 mutated AML mouse model [105].

*NPM1* mutations are typically associated with favorable prognosis, although less so in older adults. A recent retrospective study examined the response rate in patents over 65 who had either received 7 + 3, HMA alone, or HMA with venetoclax. The combination of HMA with venetoclax showed robust responses, with an OS of 80% at 1 year, and 70% at 2 years. Randomized prospective trials are now underway to assess this drug combination [106].

Other innovative drugs under investigation include cyclin-dependent kinase 9 (CDK9) inhibitors. CDK9 normally regulates transcription promoting cell proliferation and survival and is required for expression of the anti-apoptotic protein myeloid cell leukemia-1 (MCL-1). By inhibiting CDK9, the MCL-1 gene is also downregulated. Multiple CDK9 inhibitors are in preclinical or clinical development. Alvocidib has completed phase I/II clinical trials comparing the combination of alvocidib with cytarabine and mitoxantrone vs. 7 + 3, with the former showing improved CR rates over the later (70% vs. 46%; *p* = 0.003) [107]. Alvocidib is currently being investigated in R/R AML following combination therapy with venetoclax (NCT03969420), in newly diagnosed intermediate and high-risk AML in combination with 7 + 3 induction chemotherapy (NCT03298984), and in combination with decitabine in patients with MDS (NCT03593915). MCL-inhibitors have unfortunately not proven successful to date.

In addition to targeting the cell cycle, pharmacotherapies have attempted to target *RAS* oncogenes involved in the RAS-RAF-MEK-ERK pathway. This cascade controls hematopoietic stem cell growth, inhibits apoptosis and plays a role in chemoresistance. A pre-clinical study using a pan-RAF inhibitor known as LY3009120 in combination with cytarabine on AML cell lines showed synergistic effects [108]. While RAS-inhibitors have traditionally been examined in solid tumors, the promising results in pre-clinical trials may warrant further exploration in hematologic malignancies.

A list of drugs in current clinical trials is listed in Table 2. While this is not a comprehensive review of every drug currently in the pipeline, it represents some of the most exciting novel therapeutic targets being explored.

## 7. Conclusions

There is no doubt that the landscape of treating AML is becoming more and more complex. However, with this complexity comes great opportunities to find actionable mutations and develop targeted therapies that are typically oral and allow more outpatient therapy. With a plethora of new therapeutic modalities available, treatment for each individual patient will depend on a combination of the disease setting, cytogenetic and molecular profiles, functional status, prior therapies and ability to tolerate different side effects. We anticipate that within the next year, we will start to see data from pending clinical trials that will lead to even more revolutionary therapeutic options. Ideally, future studies will compare different therapies head-to-head or implement novel trial designs to test combinatorial or sequential approaches. Incorporating molecular assays and new technologies for detecting MRD, with the goal of eradication, will also hopefully nominate strategies with the highest likelihood of obtaining a cure for patients facing this devastating disease.

## Figures and Tables

**Table 1:** Recently approved therapies in acute myeloid leukemia.

Drug Name	Target	Date Approved	Approved Use	Trial	Title
Midostaurin	FLT3	1 April 2017	7 + 3 + Midostaurin in Newly Diagnosed FLT3 AML in Patients <60	NCT00651261/CALGB-RATIFY	A Phase III Randomized, Double-Blind Study of Induction (Daunorubicin/Cytarabine) and Consolidation (High-Dose Cytarabine) Chemotherapy + Midostaurin (PKC412) (IND #101261) or Placebo in Newly Diagnosed Patients <60 Years of Age with FLT3 Mutated Acute Myeloid Leukemia (AML)
Enasidenib	IDH2	1 August 2017	R/R AML with IDH2 Mutation	NCT01915498	A Phase I/II, Multicenter, Open-Label, Dose-Escalation and Expansion, Safety, Pharmacokinetic, Pharmacodynamic, and Clinical Activity Study of Orally Administered AG-221 in Subjects with Advanced Hematologic Malignancies with an IDH2 Mutation
Vyxeos	Liposomal Daunorubicin, Cytarabine	1 August 2017	AML-MRC or t-AML	NCT01696084	Phase III, Multicenter, Randomized, Trial of CPX-351 (Cytarabine:Daunorubicin) Liposome Injection Versus Cytarabine and Daunorubicin in Patients 60–75 Years of Age with Untreated High Risk (Secondary) AML
Mylotarg	CD33	1 September 2017	R/R AML with CD33 in Patients ≥2 years of age; Newly Diagnosed AML with CD33	MyloFrance 1	High Efficacy and Safety Profile of Fractionated Doses of Mylotarg as Induction Therapy in Patients with Relapsed Acute Myeloblastic Leukemia: a Prospective Study of the Alfa Group
Ivosidenib	IDH1	1 July 2018	R/R AML with IDH1 Mutation	NCT02074839	A Phase I, Multicenter, Open-Label, Dose-Escalation and Expansion, Safety, Pharmacokinetic, Pharmacodynamic, and Clinical Activity Study of Orally Administered AG-120 in Subjects with Advanced Hematologic Malignancies with an IDH1 Mutation
Gilteritinib	FLT3-ITD, FLT3- TKD	28 November 2018	R/R AML with FLT3 Mutation	NCT02421939; ADMIRAL	A Phase III Open-Label, Multicenter, Randomized Study of ASP2215 Versus Salvage Chemotherapy in Patients with Relapsed or Refractory Acute Myeloid Leukemia (AML) with FLT3 Mutation
Venetoclax	BCL2	21 November 2018	In Combination with HMA in DeNovo AML in Patients >75 Unfit for Standard 7 + 3	NCT02203773	Phase Ib Study of ABT-199 (GDC-0199) in Combination with Azacitidine or Decitabine in Treatment-Naive Subjects with Acute Myelogenous Leukemia Who Are Greater Than or Equal to 60 Years of Age and Who Are Not Eligible for Standard Induction Therapy
Glasdegib	Smoothened (SMO) receptor	21 November 2018	In Combination with LDAC in Newly Diagnosed AML in Patients ≥ 75	NCT01546038	A Study to Evaluate PF-04449913 with Chemotherapy in Patients with Acute Myeloid Leukemia or Myelodysplastic Syndrome
Oral Azacitidine	DNA methyltransferase	1 September 2020	Maintenance Therapy After Achieving First CR or CRi in Adult Patients	NCT01757535	Efficacy of Oral Azacitidine Plus Best Supportive Care as Maintenance Therapy in Subjects with Acute Myeloid Leukemia in Complete Remission (QUAZAR AML-001)

Abbreviations: AML = acute myeloid leukemia; IND = investigational new drug application; AML-MRC = acute myeloid leukemia with myelodysplasia related changes; t-AML = therapy related acute myeloid leukemia; HMA = hypomethylating agents; R/R = relapsed or refractory; CR = complete remission; CRi = CR with incomplete hematologic recovery; CALGB = Cancer and Leukemia Group B; FLT3 = FMS-related tyrosine kinase 3 gene; LDAC = low dose cytarabine; ITD = internal tandem duplications; TKD = tyrosine kinase domain; BCL2 = B-cell lymphoma 2; IDH = isocitrate dehydrogenase.

**Table 2:** Drugs in the Pipeline Newly Diagnosed AML/MDS.

Drug Name	Target	Title	Trial
Crenolanib	FLT3-ITD, FLT3-TKD	Phase III Randomized Study of Crenolanib Versus Midostaurin Administered Following Induction Chemotherapy and Consolidation Therapy in Newly Diagnosed Subjects with FLT3 Mutated Acute Myeloid Leukemia	NCT03258931
Gilteritinib	FLT3-ITD, FLT3-TKD	Open-Label, Randomized Trial of Daunorubicin/Cytarabine and High Dose Cytarabine + Gilteritinib vs. Midostaurin for Induction and Consolidation. FLT3 mutated patients will be stratified based on TKD vs. ITD. Patients who are FLT3 ITD will be further stratified by Signal Ratio (High vs. Low of FLT3 Wild Type) and Nucleophosmin 1-Mutated (NPM1) (Positive vs. Negative).	NCT03836209
Quizartinib	FLT3-ITD	A Phase III, Double-Blind, Placebo-controlled Study of Quizartinib Administered in Combination with Induction and Consolidation Chemotherapy, and Administered as Continuation Therapy in Subjects 18 to 75 Years Old with Newly Diagnosed FLT3-ITD (+) Acute Myeloid Leukemia (QuANTUM First)	NCT02668653
Midostaurin	multitarget-kinase, FLT3	A Phase I/II Study of Midostaurin (PKC412) and 5-Azacitidine for Elderly Patients with Acute Myelogenous Leukemia.	NCT01093573
Midostaurin	multitarget-kinase, FLT3	A Randomized Phase II Multicenter Study to Assess the Tolerability and Efficacy of the Addition of Midostaurin to 10-day Decitabine Treatment in Unfit Adult Acute Myeloid Leukemia and High-Risk Myelodysplasia Patients	NCT04097470
Gilteritinib	FLT3-ITD, FLT3-TKD	A Phase III Multicenter, Open-label, Randomized Study of ASP2215 (Gilteritinib), Combination of ASP2215 Plus Azacitidine and Azacitidine Alone in the Treatment of Newly Diagnosed Acute Myeloid Leukemia with FLT3 Mutation in Patients Not Eligible for Intensive Induction Chemotherapy	NCT02752035
Sorafenib	FLT3-ITD	Phase II Study of Sorafenib Plus 5-Azacitidine for the Initial Therapy of Patients with Acute Myeloid Leukemia and High-Risk Myelodysplastic Syndrome with FLT3-ITD Mutation	NCT02196857
Sorafenib	FLT3-ITD	Phase I Study of The Combination of Bortezomib and Sorafenib Followed by Decitabine in Patients with Acute Myeloid Leukemia	NCT01861314
SEL24	FLT3, PIM	A Phase I/II Study of SEL24 in Patients with Acute Myeloid Leukemia	NCT03008187
MAX-40279	FGFR, FLT3-ITD and D835	A Phase I Trial of MAX-40279 Given Orally to Subjects with Acute Myelogenous Leukemia (AML)	NCT03412292
Ivosidenib, Enasidenib	IDH1/IDH2	A Phase III, Multicenter, Double-blind, Randomized, Placebo-controlled Study of Ivosidenib or Enasidenib in Combination with Induction Therapy and Consolidation Therapy Followed by Maintenance Therapy in Patients with Newly Diagnosed Acute Myeloid Leukemia or Myelodysplastic Syndrome with Excess Blasts-2, with an IDH1 or IDH2 Mutation, Respectively, Eligible for Intensive Chemotherapy.	NCT03839771
APR-246	TP53	A Phase III Multicenter, Randomized, Open Label Study of APR-246 in Combination with Azacitidine Versus Azacitidine Alone for the Treatment of (Tumor Protein) TP53 Mutant Myelodysplastic Syndromes	NCT03745716
AMG-232	MDM2 Antagonist	A Phase IB Study of KRT-232 (AMG-232) in Combination with Decitabine in Acute Myeloid Leukemia	NCT03041688
HDM201	MDM2 Antagonist	A Phase I/II Multi-center Study of HDM201 Added to Chemotherapy in Adult Subjects with Relapsed/Refractory (R/R) or Newly Diagnosed Acute Myeloid Leukemia (AML)	NCT03760445
DS 3032b	MDM2 Antagonist	A Phase I Study of Milademetan (DS 3032b), an Oral MDM2 Inhibitor, in Dose Escalation as a Single Agent and in Dose Escalation/Expansion in Combination with 5 Azacitidine in Subjects with Acute Myelogenous Leukemia (AML) or High-Risk Myelodysplastic Syndrome (MDS)	NCT02319369
Milademetan	MDM2 Antagonist, FLT3	A Phase I Study of Milademetan in Combination with Quizartinib in Subjects with FLT3-ITD Mutant Acute Myeloid Leukemia That Are Relapsed/Refractory, or Newly Diagnosed and Unfit for Intensive Chemotherapy	NCT03552029
Venetoclax	BCL2	A Randomized, Double-Blind, Placebo Controlled Phase III Study of Venetoclax in Combination with Azacitidine Versus Azacitidine in Treatment Naïve Subjects with Acute Myeloid Leukemia Who Are Ineligible for Standard Induction Therapy	NCT02993523
Venetoclax	BCL2	A Randomized, Double-Blind, Placebo Controlled Phase III Study of Venetoclax Co-Administered with Low Dose Cytarabine Vs. Low Dose Cytarabine in Treatment Naïve Patients with Acute Myeloid Leukemia Who Are Ineligible for Intensive Chemotherapy	NCT03069352
Venetoclax + Decitabine + Quizartinib	FLT3, BCL2	Quizartinib, Decitabine, and Venetoclax in Treating Participants with Untreated or Relapsed Acute Myeloid Leukemia or High-Risk Myelodysplastic Syndrome	NCT03661307
Venetoclax + S64315	MCL1-I, BCL2	An International Phase Ib Multicentre Study to Characterize the Safety and Tolerability of Intravenously Administered S64315, a Selective Mcl-1 Inhibitor, in Combination with Orally Administered Venetoclax, a Selective BCL2 Inhibitor in Patients with Acute Myeloid Leukaemia (AML).	NCT03672695
Glasdegib	Hedgehog pathway (Hh)	A Study Evaluating Intensive Chemotherapy with or without Glasdegib or Azacitidine with or without Glasdegib in Patients with Previously Untreated Acute Myeloid Leukemia (BRIGHT AML1019)	NCT03416179
7 + 3 + midostaurin + GO	CD33, TKI	A Phase I Study to Evaluate the Safety and Preliminary Efficacy of Gemtuzumab Ozogamicin and Midostaurin When Used in Combination with Standard Cytarabine and Daunorubicin Induction for Newly Diagnosed FLT3-mutated AML.	NCT03900949
Tagraxofusp	CD123	A Phase I/II Study of SL-401 as Consolidation Therapy for Adult Patients with Adverse Risk Acute Myeloid Leukemia in First CR, and/or Evidence of Minimal Residual Disease (MRD) in First CR	NCT02270463
Alvocidib	CDK9-i	A Phase I, Open-label, Dose-Escalation, Safety and Biomarker Prediction of Alvocidib and Cytarabine/Daunorubicin (7 + 3) in Patients with Newly Diagnosed Acute Myeloid Leukemia (AML)	NCT03298984
Alvocidib	CDK9-i	A Phase Ib/II, Open-label Clinical Study to Determine Preliminary Safety and Efficacy of Alvocidib When Administered in Sequence After Decitabine or Azacitidine in Patients with MDS	NCT03593915
		**Maintenance Therapy**	
Sorafenib	FLT3-ITD	Phase II Trial (SORMAIN), a Randomized, Double-Blind, Placebo-Controlled Study, Evaluating Sorafenib as Maintenance Therapy After allo-SCT in Patients with FLT3-ITD-Positive AML.	EudraCT 2010-018539-16
Midostaurin	multi-kinase	A Phase II, Randomized Trial of Standard of Care, with or without Midostaruin to Prevent Relapse Following Allogeneic Hematopoietic Stem Cell Transplantation in Patients with FLT3-ITD Mutated Acute Myeloid Leukemia (RADIUS)	NCT01883362
Gilteritinib	FLT3-ITD, FLT3-TKD	A Multi-center, Randomized, Double-blind, Placebo-controlled Phase III Trial of the FLT3 Inhibitor Gilteritinib Administered as Maintenance Therapy Following Allogeneic Transplant for Patients with FLT3/ITD AML	NCT02997202
Enasidenib	IDH2	Pilot Trial of Enasidenib (AG-221) Maintenance Post Allogeneic Hematopoietic Cell Transplantation in Patients with IDH2 Mutation	NCT03728335
APR-246	TP53	Phase II Trial of APR-246 in Combination with Azacitidine as Maintenance Therapy for TP53 Mutated AML or MDS Following Allogeneic Stem Cell Transplant	NCT03931291
Venetoclax + Selinexor	BCL2, XPO1	An Investigator-Sponsored Phase Ib Trial of Venetoclax and SINE: Selective Inhibition of Nuclear Export in Patients with High-Risk Hematologic Malignancies	NCT03955783
Nivolumab	checkpoint inhib	PD-1 Inhibition with Nivolumab for the Treatment of Patients with Acute Myeloid Leukemia in Remission at High-Risk for Relapse	NCT02532231
		**Relapsed- Refractory AML**	
FF-10101-01	ITD, ITD-691L and D835	A First-in-Human Phase I/IIa Study to Assess the Safety, Tolerability, Efficacy, and Pharmacokinetics of FF-10101-01 in Subjects with Relapsed or Refractory Acute Myeloid Leukemia	NCT03194685
Enasidenib	IDH2	CPX-351 (Vyxeos) Plus Enasidenib for Relapsed Acute Myelogenous Leukemia Characterized by the IDH2 Mutation	NCT03825796
Idasanutlin	MDM2 Antagonist	A Multicenter, Double-Blind, Randomized, Placebo-Controlled, Phase III Study of Idasanutlin, an MDM2 Antagonist, with Cytarabine Versus Cytarabine Plus Placebo in Patients with Relapsed or Refractory Acute Myeloid Leukemia (AML)	NCT02545283
Idasanutlin	MDM2 Antagonist	A Phase IB Multi-Arm Study with Venetoclax in Combination with Cobimetinib and Venetoclax in Combination with Idasanutlin in Patients with Relapsed or Refractory Acute Myeloid Leukemia Who Are Not Eligible for Cytotoxic Therapy	NCT02670044
AMG-232	MDM2 Antagonist	A Phase IB Study of KRT-232 (AMG-232) in Combination with Decitabine in Acute Myeloid Leukemia	NCT03041688
HDM201	MDM2 Antagonist	A Phase I/II Multi-center Study of HDM201 Added to Chemotherapy in Adult Subjects with Relapsed/Refractory (R/R) or Newly Diagnosed Acute Myeloid Leukemia (AML)	NCT03760445
Milademetan + Quizartinib	MDM2 Antagonist, FLT3	A Phase I Study of Milademetan in Combination with Quizartinib in Subjects with FLT3-ITD Mutant Acute Myeloid Leukemia That Are Relapsed/Refractory, or Newly Diagnosed and Unfit for Intensive Chemotherapy	NCT03552029
ALRN-6924 wih ARA-C	MDMX	A Phase I/Ib Open-Label Study to Determine the Safety and Tolerability of ALRN-6924 Alone and in Combination with Cytarabine (Ara-C) in Patients with Relapsed/Refractory Acute Myeloid Leukemia or Advanced Myelodysplastic Syndrome with Wild-Type TP53	NCT02909972
Enasidenib + Vyxeos	IDH2	CPX-351 Plus Enasidenib for Relapsed Acute Myelogenous Leukemia Characterized by the IDH2 Mutation	NCT03825796
Quizartinib+ Vyxeos	FLT3-ITD	A Phase II Study Assessing CPX-351 (Vyxeos™) with Quizartinib for the Treatment of Relapsed or Refractory FLT3-ITD Mutation-Positive AML	NCT04209725
Venetoclax + Vyxeos	BCL2	Phase II Study of CPX-351 in Combination with Venetoclax in Patients with Acute Myeloid Leukemia (AML)	NCT03629171
Venetoclax + GO	CD33, BCL2	Phase Ib Study of the Safety and Efficacy of Gemtuzumab Ozogamicin (GO) and Venetoclax in Patients with Relapsed or Refractory CD33+ Acute Myeloid Leukemia: Big Ten Cancer Research Consortium BTCRC-AML17-113	NCT04070768
OX40	Anti-PDL1, SMO, Anti-CD33, BCL2	An Open-Label Phase Ib/II Multi-Arm Study of OX40 Agonist Monoclonal Antibody (mAb), Anti-PDL1 mAb, Smoothened (SMO) Inhibitor, Anti-CD33 mAb, Bcl-2 Inhibitor and Azacitidine as Single-Agents and/or Combinations for the Treatment of Patients with Acute Myeloid Leukemia (AML)	NCT03390296
Venetoclax + Ivosidenib	IDH1, BCL2	Phase Ib/II Investigator Sponsored Study of the IDH1-Mutant Inhibitor Ivosidenib (AG120) with the BCL2 Inhibitor Venetoclax in IDH1-Mutated Hematologic Malignancies	NCT03471260
Venetoclax + Alvocidib	CDK-9, BCL2	Phase Ib Study of Venetoclax and Alvocidib in Patients with Relapsed/Refractory Acute Myeloid Leukemia; A Phase II, Open-label, Randomized, Two-stage Clinical Study of Alvocidib in Patients with Relapsed/Refractory Acute Myeloid Leukemia Following Treatment with Venetoclax Combination Therapy	NCT03441555, NCT03969420
Venetoclax + Quizartinib	FLT3, BCL2	A Phase Ib/II Study of Venetoclax in Combination with Quizartinib in FLT3-Mutated Acute Myelogenous Leukemia (AML)	NCT03735875
Venetoclax + Decitabine + Quizartinib	FLT3, BCL2	Quizartinib, Decitabine, and Venetoclax in Treating Participants with Untreated or Relapsed Acute Myeloid Leukemia or High-Risk Myelodysplastic Syndrome	NCT03661307
Venetoclax + Gilteritinib	FLT3, BCL2	A Phase I/II Study of Azacitidine, Venetoclax, and Gilteritinib for Patients with Acute Myeloid Leukemia or High-Risk Myelodysplastic Syndrome with an Activating FLT3 Mutation	NCT04140487
Venetoclax + Gilteritinib	FLT3, BCL2	A Multicenter, Open-Label Phase Ib Study to Assess Safety and Efficacy of Venetoclax in Combination with Gilteritinib in Subjects with Relapsed/Refractory Acute Myeloid Leukemia	NCT03625505
Venetoclax + Ruxolitinib	BCL2, JAK2	Phase I Study to Evaluate Safety of Ruxolitinib in Combination with Venetoclax in Patients with Relapsed/Refractory Acute Myeloid Leukemia	NCT03874052
Venetoclax + Cobimetinib + Idasanutlin	BCL2, MEK/MDM2	A Phase IB Multi-Arm Study with Venetoclax in Combination with Cobimetinib and Venetoclax in Combination with Idasanutlin in Patients with Relapsed or Refractory Acute Myeloid Leukemia Who Are Not Eligible for Cytotoxic Therapy	NCT02670044
Venetoclax + Lintuzumab	CD33, BCL2	A Phase I/II Study of Venetoclax and Lintuzumab-Ac225 in Patients with Refractory or Relapsed AML	NCT03867682
Ivosidenib + Nivolumab	IDH1, Check point-inhibitor	A Study of the IDH1 Inhibitor AG-120 in Combination with the Checkpoint Blockade Inhibitor, Nivolumab, for Patients with IDH1 Mutated Relapsed/Refractory AML and High-Risk MDS	NCT04044209
Magolimab +/- AZA	CD47	A Phase Ib Trial of Hu5F9-G4 Monotherapy or Hu5F9-G4 in Combination with Azacitidine in Patients with Hematological Malignancies	NCT03248479
XmAv14045	CD123-CD3 BiTE	A Phase I Multiple Dose Study to Evaluate the Safety and Tolerability of XmAb^®^14045 in Patients with CD123-Expressing Hematologic Malignancies	NCT02730312
NKR-2	investigational CAR-T	A Multi-national, Open-label, Dose Escalation Phase I Study to Assess the Safety and Clinical Activity of Multiple Administrations of NKR-2 in Patients with Different Metastatic Tumor Types (THINK - THerapeutic Immunotherapy with NKR-2)	NCT03018405
CD33, CLL-1 cCAR	CD33, CLL-1	Phase I, Interventional, Single Arm, Open Label, Treatment Study to Evaluate the Safety and Tolerability of CLL1-CD33 cCAR in Patients with Relapsed and/or Refractory, High-Risk Hematologic Malignancies.	NCT03795779
DCP-001	Dendritic cells	An International, Multicentre, Open-label Study To Evaluate the Efficacy and Safety of Two Different Vaccination Regimens of Immunotherapy with Allogeneic Dendritic Cells, DCP-001, in Patients with Acute Myeloid Leukaemia That Are In Remission with Persistent MRD	NCT03697707
SNDX-5613	Menin-inhibitor	AUGMENT-101: A Phase I/II, Open-label, Dose-Escalation and Dose-Expansion Cohort Study of SNDX 5613 in Patients with Relapsed/Refractory Leukemias, Including Those Harboring an MLL/KMT2A Gene Rearrangement or Nucleophosmin 1 (NPM1) Mutation	NCT04065399
CC-95251	SIRPα antagonist/CD47	A Phase I, Open-Label, Dose Finding Study of CC-95251, A Monoclonal Antibody Directed Against SIRPα, Alone and in Combination with Cetuximab or Rituximab in Subjects with Advanced Solid and Hematologic Cancers	NCT03783403
TTI-621	CD47	A Phase Ia/Ib Dose Escalation and Expansion Trial of TTI-621, a Novel Biologic Targeting CD47, in Subjects with Relapsed or Refractory Hematologic Malignancies and Selected Solid Tumors	NCT02663518
TTI-622	CD47	A Phase Ia/Ib Dose Escalation and Expansion Trial of TTI-622 in Patients with Advanced Relapsed or Refractory Lymphoma or Myeloma	NCT03530683

Abbreviations: AML = acute myeloid leukemia; MDS = myelodysplastic syndromes; TP53 = Tumor protein p53; MDM2 = mouse double minute 2 inhibitors; CDK = cyclin-dependent kinase; MDMX = Mouse double minute X; CAR = chimeric antigen receptor; SIRP = signal regulatory protein.
